# Illusory Motion Reproduced by Deep Neural Networks Trained for Prediction

**DOI:** 10.3389/fpsyg.2018.00345

**Published:** 2018-03-15

**Authors:** Eiji Watanabe, Akiyoshi Kitaoka, Kiwako Sakamoto, Masaki Yasugi, Kenta Tanaka

**Affiliations:** ^1^Laboratory of Neurophysiology, National Institute for Basic Biology, Okazaki, Japan; ^2^Department of Basic Biology, The Graduate University for Advanced Studies (SOKENDAI), Miura, Japan; ^3^Department of Psychology, Ritsumeikan University, Kyoto, Japan; ^4^Department of Physiological Sciences, The Graduate University for Advanced Studies (SOKENDAI), Miura, Japan; ^5^Division of Integrative Physiology, National Institute for Physiological Sciences (NIPS), Okazaki, Japan; ^6^Sakura Research Office, Wako, Japan

**Keywords:** visual illusions, predictive coding, deep learning, artificial intelligence, cerebral cortex

## Abstract

The cerebral cortex predicts visual motion to adapt human behavior to surrounding objects moving in real time. Although the underlying mechanisms are still unknown, predictive coding is one of the leading theories. Predictive coding assumes that the brain's internal models (which are acquired through learning) predict the visual world at all times and that errors between the prediction and the actual sensory input further refine the internal models. In the past year, deep neural networks based on predictive coding were reported for a video prediction machine called PredNet. If the theory substantially reproduces the visual information processing of the cerebral cortex, then PredNet can be expected to represent the human visual perception of motion. In this study, PredNet was trained with natural scene videos of the self-motion of the viewer, and the motion prediction ability of the obtained computer model was verified using unlearned videos. We found that the computer model accurately predicted the magnitude and direction of motion of a rotating propeller in unlearned videos. Surprisingly, it also represented the rotational motion for illusion images that were not moving physically, much like human visual perception. While the trained network accurately reproduced the direction of illusory rotation, it did not detect motion components in negative control pictures wherein people do not perceive illusory motion. This research supports the exciting idea that the mechanism assumed by the predictive coding theory is one of basis of motion illusion generation. Using sensory illusions as indicators of human perception, deep neural networks are expected to contribute significantly to the development of brain research.

## Introduction

Deep neural networks (DNNs), which have been developed with reference to the network structures and the operational algorithms of the brain, have achieved notable success in a broad range of fields (LeCun et al., 2015; Schmidhuber, 2015), including computer vision, in which they have produced results comparable to and in some cases superior to human experts (He et al., 2015; Silver et al., 2017). When development began, the most successful DNNs for computer vision relied on supervised learning from large sets of labeled training images. However, the human brain, particularly the cerebral cortex, learns the world at least partially in an unsupervised manner (otherwise known as self-supervised learning, Hinton et al., 1995; Bengio, 2013). These theories based on unsupervised learning suggested that the brain distills the spatiotemporal structure of objects from visual information that is constantly obtained in real time and predicts the future positions and figures of the moving objects. It is assumed that the predictive ability of sensory perception interpolates inevitable neural delay and that human behaviors are adapted to the world that is progressing in real time (Nijhawan, 2008; Heeger, 2017).

**Figure 1:**
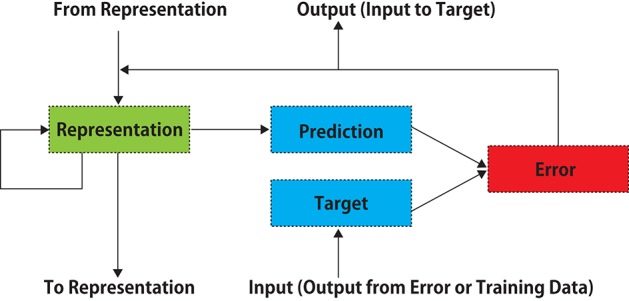
A schematic diagram of PredNet (a modification of Figure 1 in Lotter et al., 2016). Illustration of information flow within a single layer is presented. Vertical arrows represent connections with other layers. Each layer consists of “Representation” neurons, which output a layer-specific “Prediction” at each time step, which is subtracted from “Target” to produce an error, which is then propagated laterally and vertically in the network. External data or a lower-layer error signal is input to “Target.” In each layer, the input information is not processed directly, and the prediction error signal is processed.

DNNs functioning in an unsupervised learning manner similar to the cerebral cortex have been gradually developed. Using auto-encoder networks or generative adversarial networks incorporating recurrent memory cells (long-short-term-memory, LSTM), it is becoming possible to predict the future state of an object in moving images (Mathieu et al., 2015; Srivastava et al., 2015; Lotter et al., 2016; Vondrick et al., 2016; Villegas et al., 2017a,b), although only several video frames ahead. A DNN called PredNet (Lotter et al., 2016) has been intrinsically designed according to the predictive coding theory (Rao and Ballard, 1999; Friston and Kiebel, 2009; Shipp, 2016), which is one of the most influential hypotheses that can comprehensively explain the information-processing mechanism of the visual system of the cerebral cortex (see the discussion section). PredNet learns to predict future frames in a video sequence, with each layer in the network making local predictions using backward information from upper layers and forwarding only the difference values from those predictions to subsequent upper network layers (Figure 1). This artificial network is essentially comparable to the theory of brain's visual processing of predictive coding, in which backward connections from higher- to lower-order visual cortical areas carry predictive information, whereas the forward connections carry the difference values between the predictions and actual lower-level activities. It is hypothesized that the brain endeavors is to minimize such difference values (Rao and Ballard, 1999; Friston and Kiebel, 2009; Shipp, 2016).

**Figure 2:**
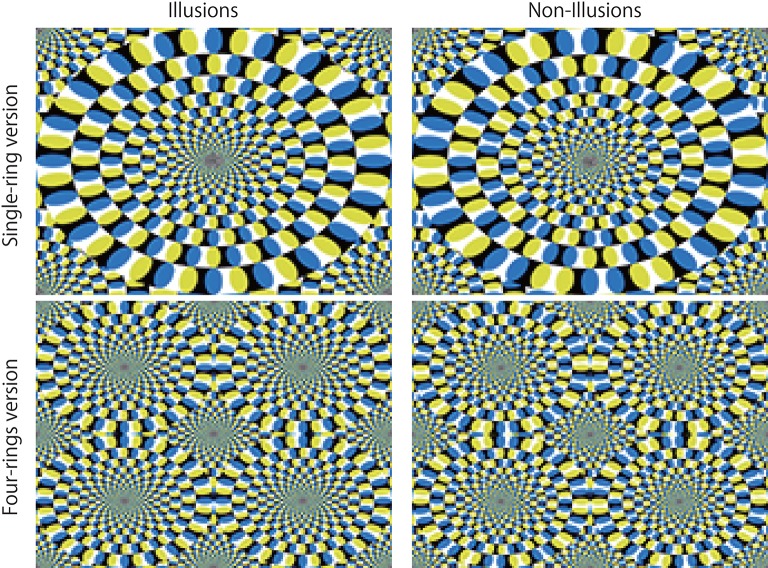
Akiyoshi Kitaoka's rotating snake illusions (the left 2 panels). In the case of the single-ring version, most people perceive a counter-clock wise motion. In the case of the four-ring version, people perceive clockwise or counter-clockwise motion depending on color alignment. Negative controls (non-illusions) for which people perceive no motion are presented in the right 2 panels. The resolution of all images is width 160 and height 120 pixels. For prediction, the same consecutive 20 images were input to the trained networks. To experience stronger illusory motion perception, please refer to “Akiyoshi's illusion pages,” http://www.ritsumei.ac.jp/~akitaoka/index-e.html.

While human visual prediction is extremely accurate, “mistakes” are occasionally made, such as in the case of visual illusions. For example, motion illusion is one of the visual illusions in which we perceive motion that is different from that of the physical stimulus. In the most prominent case represented by the rotating snake illusion (Figure 2), the perception of motion arises from a completely static image (Kitaoka and Ashida, 2003; Conway et al., 2005). Despite being a still image, the rotating snake illusion induces strong perception motion in humans, cats (Bååth et al., 2014), and even fish (Gori et al., 2014). Concerning this notable static stimulus, neurological and psychological studies suggest that both ocular motion and the information processing of the cerebral cortex are responsible for the perception of illusory motion (Hisakata and Murakami, 2008; Kuriki et al., 2008; Ashida et al., 2012). Predictive coding theory has been suggested to be a theoretical mechanism to generate illusions (Notredame et al., 2014; Nour and Nour, 2015; Raman and Sarkar, 2016; Shipp, 2016), including several motion illusions (Watanabe et al., 2010; Edwards et al., 2017). If the predictive coding theory explains these illusions, one might except that PredNet would also include “mistakes” similar to those associated with visual illusions for human.

It is worth considering whether indicators such as visual illusions can be reproduced in DNNs as models of the brain. The visual illusions that have been used to analyse the mechanism of visual processing in ordinary brains and to study psychiatric disorders (Gori et al., 2016) may contribute to the study of DNNs as models of the brain. As another viewpoint, DNN technologies are now being applied in the real world. To understand the risks of DNNs, it is therefore critical to know whether DNNs would be misled by the same visual illusions as humans. The visual illusion reflects the constraints operating in the visual system to support the valid formation of visual representations of our external environment and may be a legitimate adaptation to our living environment (Eagleman, 2001); however, such misperception could constitute a fatal mistake, depending on the application of DNNs.

For both purposes, videos of self-motion of viewers were input to PredNet to allow it to learn the spatiotemporal structure of the world via unsupervised learning. We then investigated whether the trained networks could predict a simple rotational motion of a propeller. Next, the rotating snake illusion, which PredNet had not experienced, was input to the trained networks, and we examined whether the prediction image contained an illusionary motion element.

## Methods

### Learning videos and generating predicted images by DNN

**Figure 3:**
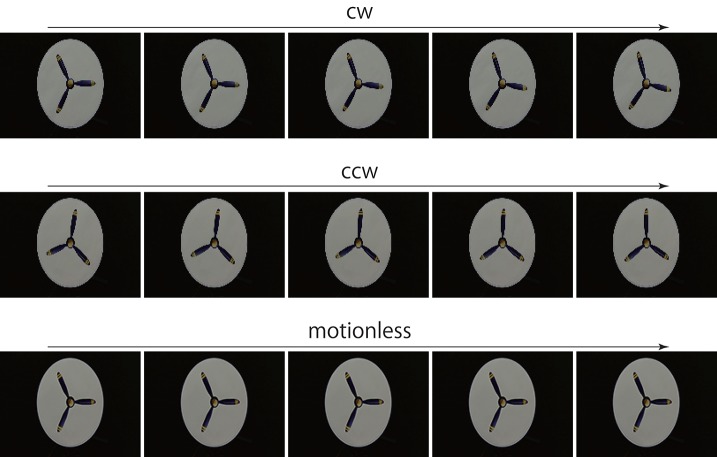
Rotating propeller. For prediction, a series of 20 consecutive images was input to the trained networks. The first 5 consecutive images are presented here. Resolution of all figures is width 160 and height 120 pixels. The original videos are available at https://doi.org/10.6084/m9.figshare.5483680.v1.

PredNet (Lotter et al., 2016), written in Keras (Chollet, 2015), was ported to Chainer (Tokui et al., 2015), and reconstructed for convenience (https://doi.org/10.6084/m9.figshare.5483710). According to previous results (Lotter et al., 2016) of a random hyperparameter search for learning of a natural image sequence, a four-layer model with 3 × 3 filter sizes for all convolutions and stack sizes per layer of 3, 48, 96, and 192 was adopted. Model weights were optimized using an Adam algorithm (Kingma and Ba, 2014) with default parameters. Models were trained with mean-squared error using videos from the First-Person Social Interactions Dataset (Fathi et al., 2012), which contains day-long videos of eight subjects spending their day at Disney World Resort in Orlando, Florida. The cameras were mounted on a cap worn by the subjects. Eight randomly selected videos from five subjects (Alin 1 and 2, Denis 1, Hussein 1, Michael 2 and 3, and Munehiko 2 and 3) were down-sampled and formatted to MP4 (width of 160 × height of 120 pixels, 30 fps, https://doi.org/10.6084/m9.figshare.5483668.v1) and were reformatted to serial still JPEG images (160 × 120 pixels). In total, the training set consisted of ~530 K images. Each image set derived from eight videos was sequentially input to the network. Model was updated every 20 consecutive images (batch) by backpropagation using the last prediction error of batch. In an experiment (**Figure 5**), mirrored (horizontally inversed) images were used for training. Training lasted ~10 h on a GPU (GTX-1080, NVIDIA). For the PredNet predictions, there were six testing stimuli: cw rotating propeller, ccw rotating propeller, static propeller, mirrored cw rotating propeller (becomes ccw), mirrored cw rotating propeller (becomes ccw), and rotating snake illusion. In each case, the stimuli were made as 20 frame sequences and the first three PredNet predictions were defined as P1, P2, and P3. The trained network predicted the 21st image (P1) with reference to 20 consecutive images (T1 to T20). Next, it predicted the 22nd image (P2) with reference to 21 consecutive images (T1 to T21) using P1 as the image of T21. The same was the case with P3. Test images (JPEG format, 160 × 120 pixels) were down-sampled from videos of rotating propellers (Figure 3, clockwise and counter-clockwise at 15 rpm, 1280 × 720 pixels, 30 fps) and sampled from the rotating snake illusions (160 × 120 pixels)(https://doi.org/10.6084/m9.figshare.5483680.v1). The program for prediction is incorporated in the above Chainer program for training. At the time step after inputting 20 image sequences of the above-mentioned test stimuli, the activation patterns of the hidden units of PredNet layers were visualized. Visualization was performed using a self-made converter program (https://doi.org/10.6084/m9.figshare.5483710.v1) and TensorBoard in TensorFlow (Version 1.2.1) (Abadi et al., 2016). As a reference for the discussion, we have provided the activation patterns of two particular units out of 181 hidden units (**Figures 11**, **12**).

### Optical flow analysis

Motion vectors observed between two consecutive predicted images (P1/P2 or P2/P3) were measured through optical flow analysis, in which optical flow vectors were calculated by the Lucas-Kanade method (Lucas and Kanade, 1981) using a customized Python program (https://doi.org/10.6084/m9.figshare.5483716.v1, window size 50, quality level 0.3). The rotation center of the propeller was calculated from the motion picture of the propeller by the ImageJ Java program (Schneider et al., 2012). The provisional rotation center of the rotating snake illusion was taken as the center of the ring. The coordinates of the calculated optical flow vectors were transformed into the coordinate system of the original videos or the still images, and the angular velocity was then calculated from the transformed coordinates and the provisional rotation center. The angular velocities calculated from optical flow vectors were averaged for each consecutive pair of the predicted images. A positive sign was assigned for cw rotation. To analyse the differences based on the type of input images, values from 400 to 1000K (number of images) trained networks were averaged (**Figure 6**), since the illusory motion predicted by the trained networks was observed over 400 K in trained networks (**Figure 8**). Significant differences between the mean values were analyzed using unpaired *t*-tests (one-tailed Welch's *t*-test or Student's *t*-test).

## Results

**Figure 4:**
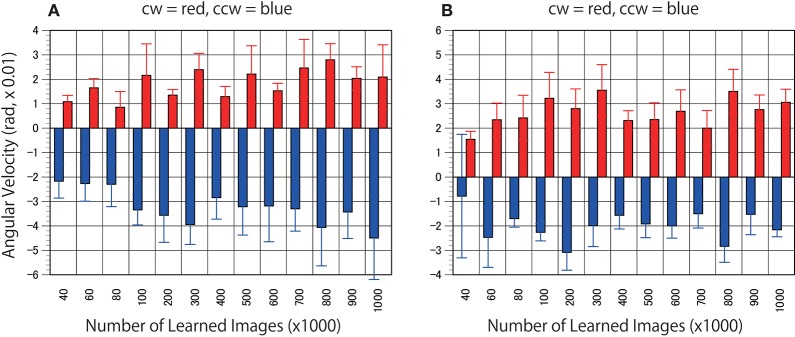
Predicted motion found in the rotating propellers. **(A)** Optical flow vectors detected between a pair of the first/second consecutive predictive images (P1/P2) of the rotating propellers (15 rpm). **(B)** Mirrored images of the rotating propellers were used as test stimuli. In other words, the cw of A and the ccw of B are derived from the same video, and the ccw of **(A)** and the cw of (**B)** are derived from the same video. Six optical flow vectors were detected in each pair of predicted images. Mean angular velocity was calculated from the optical flow vectors. A positive sign was assigned to clockwise rotation. The results of the clockwise propeller are presented in red, and the results of the counter-clockwise propeller are presented in blue. The rotation rate of 15 rpm is approximately comparable to 5.23 rad (× 0.01). Error bars indicate standard errors, and the slanted zero horizontal lines appear to a kind of café wall illusion.

**Figure 5:**
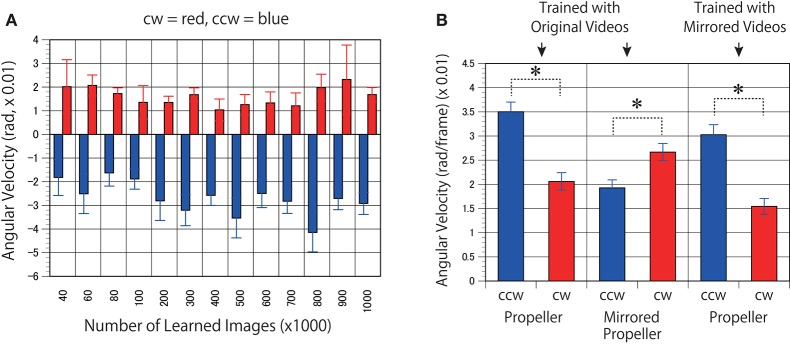
Models trained with mirrored videos also predicted motion in the rotating propellers. **(A)** The DNNs were trained using mirrored images of the videos of self-motion of viewers. Predicted angular velocities of propellers were calculated as in Figure 4A. **(B)** Mean angular velocities (P1/P2) derived from 400 to 1000 K trained networks were averaged (*n* = 7) and set to absolute values. Error bars indicate standard errors. Asterisks indicate a significant difference (*p* < 0.01).

**Figure 6:**
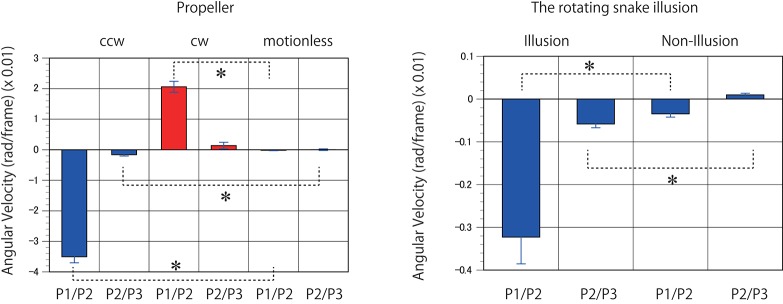
Predicted motion detected in the rotating propellers and the illusions. Mean angular velocities derived from 400 to 1000 K trained networks were averaged (*n* = 7). Error bars indicate standard errors. Asterisks indicate a significant difference (*p* < 0.01).

**Figure 7:**
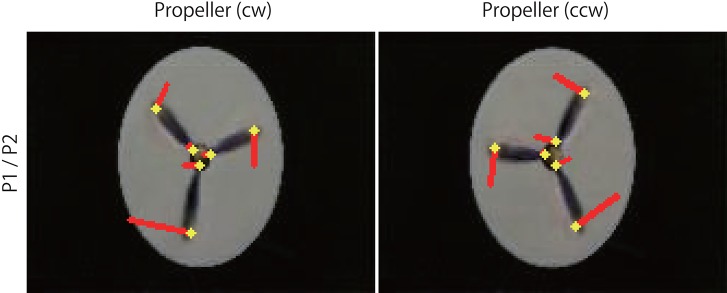
Optical flow vectors detected in the rotating propellers. The vectors were obtained between a pair of the first/second consecutive predictive images (P1/P2) of the rotating propellers. Red bars denote the direction and magnitude of vectors, yellow dots denote the start points of the vectors. To aid visualization, the magnitude of the vectors was amplified 30 times. The vectors were written over the P2 images (width 160, height 120 pixels). A network trained with 500 K video frames was used for prediction.

First, to determine whether the trained networks of PredNet are capable of predicting the real motion of an object, videos of propellers rotating at 15 rpm (Figure 3, clockwise [cw] and counter-clockwise [ccw]) and 0 rpm (Figure 3) were input to the trained networks and used to produce three consecutive predicted images (P1, P2, and P3). Using optical flow analysis, the motion vectors generated between two consecutive predicted images (P1/P2, P2/P3) were quantified, and the angular velocity with the center of propeller rotation was calculated. Figure 4A presents the magnitude of the detected angular velocity of P1/P2. The network models trained with more than 40 K training images predicted rotational motion for both cw and ccw direction, and the direction of the angular velocity corresponding to the direction of propeller rotation was extracted (Supplementary Movies). The predicted rotation motion of P1/P2 was significantly stronger in the ccw direction than in the cw direction (Figure 5B, *p* < 0.01, *t* = 4.95, degrees of freedom = 12, comparing average absolute values). Using mirrored images of the rotating propellers, a similar experiment was conducted (Figure 4B). Results similar to those of the experiment using the original image were obtained, but the predicted rotation motion of P1/P2 was significantly stronger in the cw direction than in the ccw direction (Figure 5B, *p* < 0.01, *t* = 2.82, degrees of freedom = 12, comparing average absolute values). In experiments wherein training was performed with a mirror image set (Figure 5A), the pattern of asymmetry did not change (Figure 5B, *p* < 0.01, *t* = 5.17, degrees of freedom = 12, comparing average absolute values). The propeller had a subtle asymmetrical shape along the direction of rotation. Therefore, the trained DNNs appeared to respond sensitively to the subtle differences. The magnitude of the optical flow vectors close to the rotation center was smaller than that at the periphery, which reflects the relative moving distance observed in each part of the propeller in the video (Figure 7). The rotational motion also existed in P2/P3 but became extremely small (Figure 6). In the cases of P2/P3, a significant difference was detected only between ccw and 0 rpm (*p* < 0.01, *t* = −2.951, degree of freedom = 12) and not between cw and 0 rpm (*p* = 0.156, *t* = 1.088, degrees of freedom = 12). The smaller effect observed in P2/P3 is simply due to the fact that P2/P3 started predicting from a frame that was temporally latter than P1/P2. In the original description of PredNet (Lotter et al., 2016), the test videos used for prediction were the same type of videos used for learning (e.g., car-cam video vs. car-cam video). In this study, ~5 h of videos of viewer self-motion were input to PredNet for learning without supervision of the rotating propeller. PredNet was still capable of predicting not only the shape and colors of the propellers but also the direction of propeller rotation, indicating that PredNet successfully generalized the spatiotemporal structure of moving objects as predictive information within the network.

**Figure 8:**
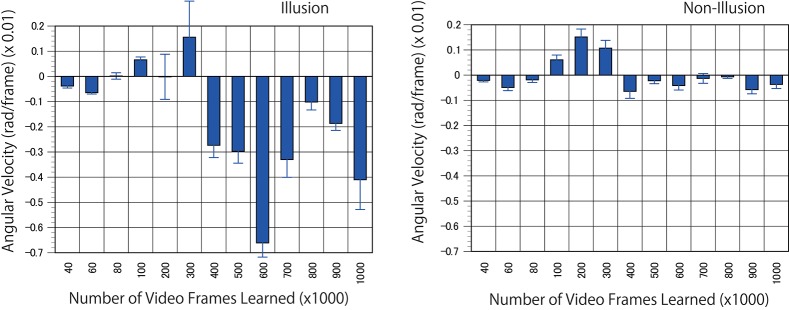
Predicted motion found in the illusion. Optical flow vectors detected between a pair of consecutive predictive images (P1/P2) of the illusion. The mean angular velocity was calculated from the optical flow vectors. A positive sign was assigned to clockwise rotation. One hundred optical flow vectors were detected in each pair of images. Error bars indicate standard errors. Although the reason was unknown, unidirectional optical flows were observed in a wide area of the predicted images from 100 to 300 K. The magnitude of optical flows was biased by location. The apparent positive angular velocities from 100 to 300 K appeared to be caused by the bias.

**Figure 9:**
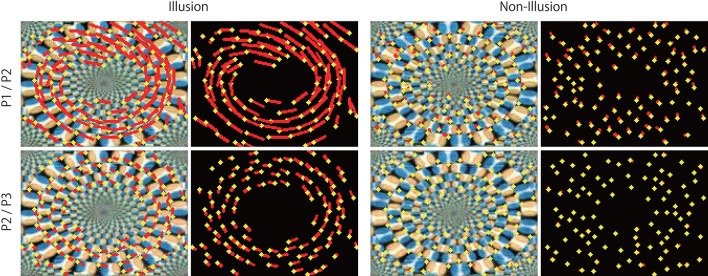
Optical flow vectors detected in the illusion. Optical flow vectors detected between a pair of consecutive predictive images (P1/P2 or P2/P3) of the illusion. The magnitude of the vectors was amplified 60 times. The left is a single ring of the rotating snake illusion, and the right is a negative control image. The vectors were imposed over the P2 or P3 images or black background (width 160, height 120 pixels). A network trained with 500 K video frames was used for prediction.

For the next predictive trained networks generated, 20 serial pictures (repeating files of the same still images) of the rotating snake illusion were input into the trained networks with output prediction for three consecutive images. The optical flow analysis revealed that rotational motion was detected on the images generated by the trained networks when ~400 K video frames or more were learned (Figures 8, 9, and Supplementary Movies). To generate rotational motion for pictures of an illusion, longer training times were required than for the rotation prediction of the propeller. The results suggested that the performance of hidden units involved in the reproduction of the illusory motion continued to be refined during training with 40 to 300 K images. It is an interesting question whether hidden units involved in the illusory motion have emerged as refinements of hidden units related to propeller rotation, or whether they emerged independently as different hidden units from propeller rotation. It will be useful to analyse the time series change of the characteristics of the hidden units.

**Figure 10:**
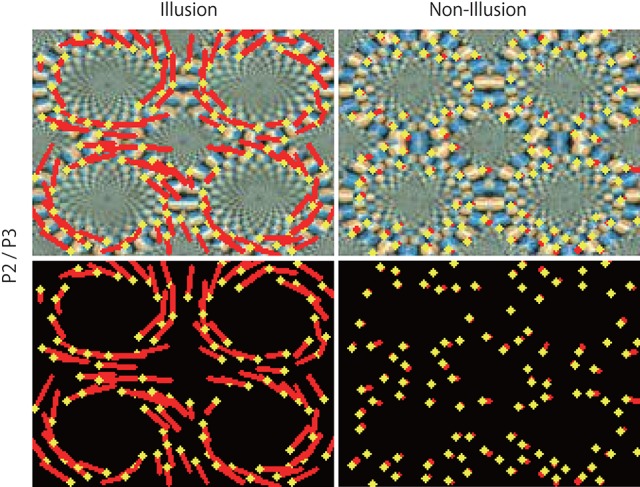
Predicted motion in two rotation directions in the illusion. Optical flow vectors detected between consecutive predictive images (P2/P3) of the illusion (**Left**). **Right** panels are the non-illusion control. The vectors were imposed over the P3 image or black background. The magnitude of the vectors was amplified 180 times. A significantly larger value (0.081 ± 0.0017) was observed in the illusion (*p* < 0.01, *t* = 37.53, degree of freedom = 127) than in the non-illusion control (mean magnitude of optical flow vectors, 0.013 ± 0.00064 pixels). The mean of magnitudes (0.080 ± 0.0025) of the vectors detected by the counter clockwise rings of the illusion was not significantly different from the value (0.081 ± 0.0023) of the clockwise rings (*p* = 0.390, *t* = −0.281, degree of freedom = 98).

The direction of the rotational motion predicted for the illusion was in agreement with the rotation direction of illusory motion perceived by humans (Figures 8, 9). When negative control static images (Figures 8, 9) containing the same shape and color sequence (blue-black-yellow-white or white-yellow-black-blue) as the rotating snake illusion for which humans do not perceive illusory motion were input to the trained networks, rotational motions other than small optical flows were not predicted for the negative controls (Figures 8, 9). When the rotating snake illusion including four illusionary rings in a single image was input to the trained networks, rotating motion was detected in all four rings in the predicted images in the cases of P1/P2 and P2/P3 (Figure 10). The cw and ccw rotating motion were detected simultaneously in the single image, which coincided again with the rotation direction perceived by humans. In the case of four rings experiment (Figure 10), the detected optical flows were concentrated to peripheral region of a ring as compared with the one ring experiment (Figure 9). Regardless of whether it was an image of one ring or an image of four rings, a single image was composed of 160 width × 120 height pixels. In other words, in the case of the four rings, one ring was composed of only 40 width x 30 height pixels. The central region of a ring of the rotating snake illusion was drawn with smaller-sized basic elements than the peripheral region. Therefore, we assumed that the generation of the predicted illusory motion depends on the size of the basic elements. In the case of the four rings in the negative control still images, only a small optical flow that differed from rotation was detected (Figure 10).

## Discussion

The DNN that predicted the motion vectors of the unlearned rotating propeller also predicted the rotational motion in the rotating snake illusion, in manner similar to human perception. Previous fMRI studies demonstrated that the rotating snake illusion activated V1 to MT areas of the cerebral cortex (Kuriki et al., 2008; Ashida et al., 2012), which are commonly activated when detecting actual moving objects and global movement in the background. In order to compare the current results to those based on knowledge of the physiology of the human brain, the DNNs were oversimplified, but it is noteworthy that the DNNs based on a theory of the cerebral cortex reproduced illusory motion in a manner similar to human perception. However, although the current experimental results were notable, they did not accurately reproduce the illusory motion perceived by humans. For instance, since illusory rotation of a ring wherein human direct attention is not induced, four simultaneous rotations of the rings presented in Figure 10 are extremely unlikely to occur in a similar manner as human perception (Hisakata and Murakami, 2008). Moreover, the speed of the rotational motion perceived in each ring varies from moment to moment. It may be better to think of PredNet as an abstractive reproduction of a small part of the biological aspects of visual information processing.

In order to verify these phenomena by DNNs, it is at least necessary to reproduce the functional division of the central visual field with a high resolution and the peripheral visual field with a low resolution. Attempts have also been made to incorporate the mechanism that distinguishes between a central visual field and a peripheral visual field into DNNs (Wang and Cottrell, 2017). An application of the DNN will be interesting as a future subject. As mentioned in the introduction section, various methods have been devised for deep learning machines that conduct video prediction (Mathieu et al., 2015; Srivastava et al., 2015; Lotter et al., 2016; Vondrick et al., 2016; Villegas et al., 2017a,b), including CNN-LSTM Encoder-Decoder used as a control in the original paper of PredNet (Lotter et al., 2016). By analyzing the DNNs other than PredNet, we will be able to deepen understanding of the mechanism by which the illusory motion is predicted.

Nevertheless, it is remarkable that the DNN “perceives” the parameters of the physical world as distorted. The information processing of the brain generates illusions not only on visual motions but also on many other parameters, such as the brightness, positions, shapes, colors, sizes, and so on. Determining that the DNN experiences these illusions requires additional investigation, but to use DNN technology in the real world introduces the possibility that these illusions could create errors associated with substantial risks. Users must be aware of this possibility.

The cerebral cortex related to vision is divided into multiple functional areas. Connection between these areas is not a one-way neural network from V1, which is close to the input source of the senses to higher-order areas, but is a reciprocal neural network that allows information to flow in both directions (Friston, 2005; Muckli and Petro, 2013). Based on the anatomical and physiological knowledge derived from primates, a basic theory of predictive coding was proposed from the viewpoint of computation theory (Kawato et al., 1993). The theory postulated that the higher-order regions of the cortex encoding the inverse model of the visual world transmit the prediction signal toward the lower-order regions and that each region detects the prediction error in reference to the sensory signals derived from the input source. Later, this theory was widely applied in the field of neuroscience (Rao and Ballard, 1999; Friston and Kiebel, 2009) and has been also developed as “free energy theory,” which is based on a variational Bayesian method (Friston and Kiebel, 2009; Bogacz, 2017). The concept can be applied to more than just the cerebral cortex. For example, it has been hypothesized that the cerebellum performs predictive learning in association with the cerebral cortex (cerebro-cerebellar loops) and seems to contribute not only to sensory motor control but also to attention, language, social cognition, and other functions (Butcher et al., 2017; Sokolov et al., 2017). Although it may not be strictly predictive coding, the dopamine signal originating from the midbrain is thought to code “prediction errors of reward learning,” and a similar learning algorithm to that of the cortex seems to be used even if the time from the start of the behavior to the result is relatively long (Schultz, 1998; Schultz and Dickinson, 2000; Keiflin and Janak, 2015; Diederen et al., 2017; Nasser et al., 2017). Therefore, although PredNet was made for engineering purposes, it is expected to become one of the key tools in the future for studying the operational principles of the brain. The predictive coding theory was also expected to be a theoretical mechanism to generate illusions (Notredame et al., 2014; Nour and Nour, 2015; Raman and Sarkar, 2016; Shipp, 2016) and was used as an explanation for certain motion illusions (Watanabe et al., 2010), of bi-stable perception (Weilnhammer et al., 2017) and of an apparent motion illusion (Edwards et al., 2017). The present result reveals the exciting idea that the predictive coding theory accounts for a wide range of visual illusions.

**Figure 11:**
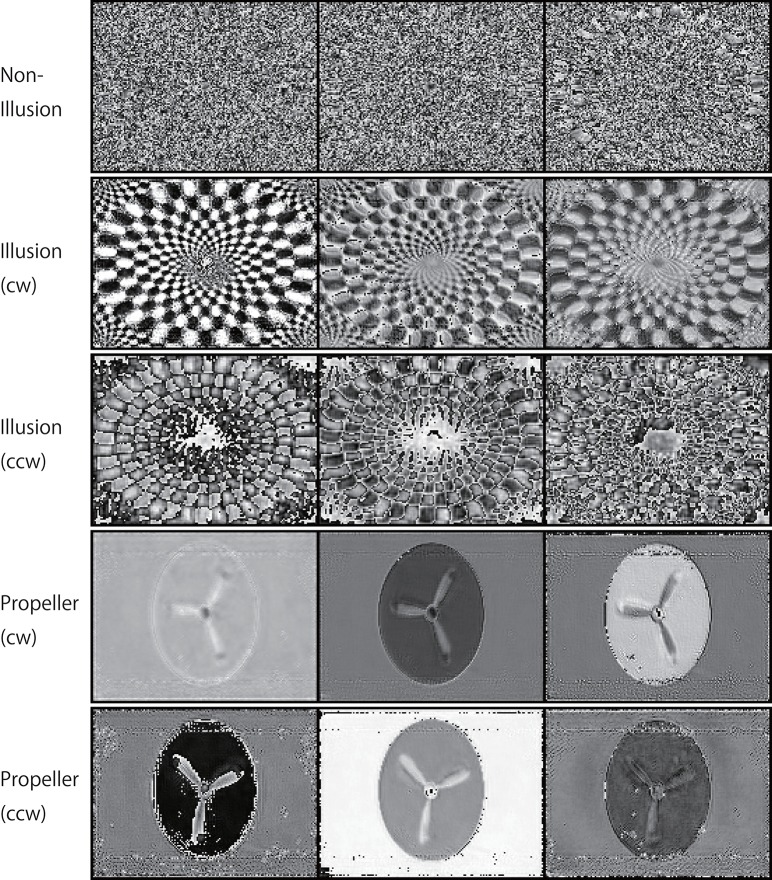
Activation patterns of the hidden units of DNNs can be visualized (a lower layer). Samples of the activation patterns of receptive fields in a lower convolution LSTM layer are presented (R-Layer0/ConvLSTM/Variable_1_3_120_160_float32_29, refer to image data numbers of TensorBoard). A network trained with 500K video frames was used for prediction. Twenty consecutive images were input to the trained networks, and visualization was then performed using TensorBoard. In this layer, there are three channels with a 120 (width) × 160 (height) pixel image. In the cw illusion, horizontal inversion pictures of the single-ring illusion (ccw) presented in Figure 2 were used as input pictures. Note that noisy random patterns are specifically observed in “Non-Illusion”.

**Figure 12:**
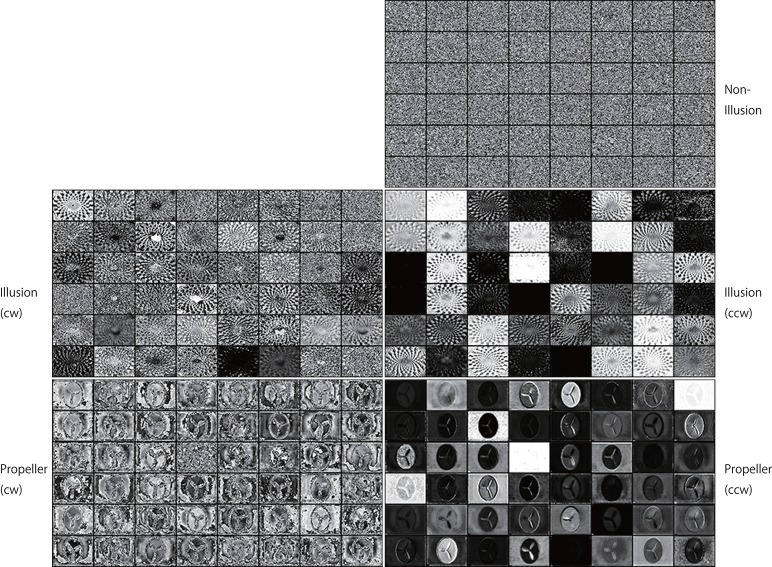
Activation patterns of the hidden units of DNNs can be visualized (an upper layer). Samples of the activation patterns of receptive fields in an upper convolution LSTM layer are presented (R-Layer1/ConvLSTM/Variable_1_48_60_80_float32_30, refer to image data numbers of TensorBoard). A network trained with 500 K video frames was used for prediction. Twenty consecutive images were input to the trained networks, and visualization was performed by TensorBoard. In this layer, there are 48 channels with a 60 (width) × 80 (height) pixel image. Note that noisy random patterns are specifically observed in “Non-Illusion,” and that a different pattern appears between the cw and ccw groups.

Since the learning methods of DNNs are visualized as mathematical formulas and program codes and the resulting trained networks can be observed (Figures 11, 12, for example), DNNs can be expected to be powerful tools for verifying the theories and hypotheses proposed in the research fields of neuroscience and psychology (Kriegeskorte, 2015; Cichy et al., 2016; Marblestone et al., 2016; VanRullen, 2017). Detailed comparisons between the biological brain and DNNs with the supervised learning method have been performed. In the context of object recognition, stimulus representations developed by the DNNs have been shown to account for neural signals in primates inferior temporal cortex and in fMRI recording data from the human ventral stream (Cadieu et al., 2014; Khaligh-Razavi and Kriegeskorte, 2014; Güçlü and van Gerven, 2015). Several DNNs were used as computational models for human shape sensitivity; the output layers of the DNNs successfully developed representations that closely related human shape judgement (Kubilius et al., 2016). These reports suggested that some fundamental processes that are shared across different hardware have been captured by DNNs. Nevertheless, DNNs and the brain are considerably different. The hardware components are completely different, and computational algorithms that are not found in the brain, such as back-propagation, are used for DNNs. DNNs manufactured for engineering purposes tend to advance their own evolution rather than remaining close to the biological brain. Only by focusing on physiological knowledge will it be possible to keep the DNN technology in the research area of the biological brain. The present research result suggests that neuroscientists and psychologists should not underestimate the value of the method (VanRullen, 2017) to call “Reverse Psychology.” Various types of sensory illusions represented by visual illusions, at least, could be the lodestar that supports the validity of DNNs as a tool for studies of the brain.

## Author contributions

EW conceived the research and designed the project. AK provided the illusory illustrations and inspired the project members. KT wrote all Python programs. EW and KS collected videos, ran programs, and measured the optical flow data. EW and MY performed data analysis. EW wrote the manuscript. All authors discussed the results and commented on the manuscript.

## Conflict of interest statement

KT was employed by company Sakura Research Office. The other authors declare that the research was conducted in the absence of any commercial or financial relationships that could be construed as a potential conflict of interest.

## Supplemental movies

A pair of consecutive predictive images was combined in AVI movie format. A network trained with 500K video frames was used for prediction.

*Propeller_L.avi*: Predictive images of the ccw-rotating propeller.

*Propeller_R.avi*: Predictive images of the cw-rotating propeller.

*Propeller_Z.avi*: Predictive images of the motionless-rotating propeller.

*Illusion_single.avi*: Predictive images of the four rings non-illusion.

*Non_illusion_four.avi*: Predictive images of the single ring illusion.

*Illusion_four.avi*: Predictive images of the four rings illusion.

*Non_illusion_single.avi*: Predictive images of the single ring non-illusion.

The movies were uploaded at https://doi.org/10.6084/m9.figshare.5483689.v1.
